# Effect of attention bias modification on depressive affect

**DOI:** 10.1038/s41598-025-09374-4

**Published:** 2025-07-11

**Authors:** Nazende Öksüz Özdemir, Serhat Yüksel

**Affiliations:** Department of Psychology, Doğuş University, Dudullu Osb Mah. Nato Yolu Cad. 265, 1 Ümraniye, Istanbul, Turkey

**Keywords:** Depressive affect, Attention bias, Cognitive bias, Cognitive bias modification

## Abstract

Cognitive interventions targeting depressive mood and major depressive disorder have emerged as a critical focus in mental health research. While studies on anxiety disorders, social phobia, eating disorders, and addiction have consistent findings, research on attentional bias modification in depression yields conflicting results depending on factors such as the severity of depression, the type and content of the modification, sample characteristics, and research design. Therefore, this study investigates the effects of attentional bias modification (ABM) on depressive mood and attentional bias levels using a randomized controlled design. In this study, a total of 45 volunteer participants, aged 18–40, were randomly assigned to experimental, placebo, and non-intervention groups. Pretest and posttest measurements of participants’ depression levels were taken using the Hamilton Depression Scale and the Mood-State Adjective Pairs List, while attentional bias was assessed using the Dot Probe Task. The results revealed statistically significant differences in the levels of attentional bias and depressive mood in the experimental group (F(2, 42) = 6.73, *p* < 0.05, η^2^ = 0.24). A significant difference was found between the pre-test and post-test scores on the Mood-State Adjective Pairs List in the experimental group, but no such effect was observed in the control groups (F(2,42) = 6.12, *p* = 0.005, η^2^ = 0.23). In the Dot Probe Task results, the experimental group showed a quicker improvement in reaction time to the positive stimulus compared to the placebo group (F(2, 42) = 5.18, *p* = 0.01, η^2^ = 0.20). The findings of this study highlight the effectiveness of attentional bias modification in significantly reducing both attentional bias and depressive mood levels in the experimental group, underscoring its potential as a powerful intervention for alleviating depression.

## Introduction

Depression is a mood disorder characterized by a diminished interest in activities that were once pleasurable, a decline in self-esteem, persistent sadness, reduced mental and physical energy, disturbances in sleep and appetite, restricted thought patterns, slower cognitive functions, and an overwhelming sense of distress^1^. While scientific studies on interventions for depression and depressive affect and the etiology of depression have made progress, depression and depressive affect remain prevalent public health issues. The development of major depression, which involves long prodromal periods, highlights the importance of preventive interventions during this phase^2–4^. Moreover, various interventions targeting residual symptoms, which are known to have a negative impact on functionality, are likely to be the subject of future research^5^. Therefore, this study investigates the effect of attentional bias modification on depressive affect and attentional bias levels.

### Depressive affect and attentional bias modification

Researchers investigating the etiology, persistence, and relapse of depression highlight the role of cognitive biases^6^. Types of biases in cognitive processes such as attention, interpretation, memory, and cognitive control influence emotional states and coping strategies, thereby laying the foundation for the development of various psychopathologies^7^. The main cognitive bias models include Beck’s “Schema Model”^8^, Ingram’s “Information Processing Analysis”^9^, Williams et al.^10^ “Impaired Cognitive Control Hypothesis”, and the Combined Cognitive Bias Model^11^.

According to Beck’s Schema Model, critical early experiences shape the schemas at the core of cognitive structures. These experiences can be singular events or recurring experiences that influence an individual’s psychological makeup. Schemas form the basis of dysfunctional assumptions about ourselves, others, the world, and the future^8^. These assumptions are the source of automatic thoughts that arise in encountered situations. The behavioral, motivational, emotional, cognitive, and somatic components of depression emerge with the activation of these schemas. It is not possible for an individual without maladaptive schemas to develop depression. Longitudinal studies conducted with children and adolescents support this information. For example, in a 16-month study, it was found that the maladaptive schemas of 572 adolescents with an average age of 15.78 were associated with bullying victimization and depressive symptoms^12^. Beck emphasizes that attentional, memory, and interpretative biases in depression are related to these schemas. These biases, combined with limited self-rewarding and self-punishing tendencies, play a significant role in the development of depression and its recurrence after treatment^8,13,14^.

Ingram’s “Information Processing Analysis” theory, while similar to Beck’s theory in emphasizing the impact of cognitive processes in depression, evaluates these processes across three different domains: network theories, affective structures, and the depth of processing. Network theories argue that past experiences are stored in memory as nodes based on conceptual similarities, and these nodes are activated by environmental or internal stimuli^9,15^. Affective structures highlight that some nodes in the memory network consist of concepts related to emotions, and these nodes interact with thought networks^16,17^. The depth of processing suggests that the semantic characteristics of stimuli and their relationship with the self-ensure detailed analysis of information, making it more likely to be stored in memory^9,18^. This theory provides an important explanation by addressing the functioning of cognitive processes in depression from different perspectives.

Williams et al.^10^ developed a cyclical model explaining the increased encoding of cues consistent with the memory network and their retrieval with new stimuli^10^. According to this model, called the “Cognitive Framework,” attention and interpretation processes strengthen cognitive representations, making them more accessible, while the elaboration phase refers to the strategic processes that shape these representations. The affective decision-making mechanism and resource allocation mechanism play a critical role in the formation of negative bias. This process begins with the redirection of attention toward negative stimuli. According to Information Processing Analysis, depressed individuals process external stimuli with biased and deep cognitive analysis, activating a memory network composed of depressive information. This network not only activates but also strengthens, preparing the ground for future cognitive biases. Negative stimuli are processed more during the attention process, creating a disruptive effect in working memory. The depressive cognitive structure becomes solidified through frequent activation, leading to the emergence of ruminative thoughts even in non-emotional tasks, which continues to negatively impact daily life^9,19^.

The “Impaired Cognitive Control Model” defines cognitive control in depression as the inability to inhibit contextually irrelevant stimuli and the difficulty in directing attention to stimuli that are appropriate for task-relevant goals^20^. This control encompasses executive functions such as inhibition, working memory, and cognitive flexibility. Cognitive flexibility refers to the ability to process stimuli impartially and develop new coping strategies, and it varies depending on psychological resources^21,22^. Depressed individuals have difficulty limiting the access of negative information to working memory and removing negative content. This results in the storage of negative information in long-term memory, the persistence of rumination, and difficulties in emotional regulation^11,23,24^.

According to the Combined Cognitive Bias Model, attentional and interpretive biases reinforce each other, playing a critical role in the development of depression. The difficulty in inhibiting negative stimuli and the inability to update information in working memory also hinders the processing of positive information^25^. Research has shown that, especially in dysphoric individuals, attention processes trigger interpretive and memory biases^26^. A study with children and adolescents found that recall memory and interpretive biases were associated with depressive symptoms, and that multiple biases had a strong preparatory effect on depression^27^. Cognitive bias modification methods are divided into “Cognitive Bias Modification-Attention” and “Cognitive Bias Modification-Interpretation”^28^. Attention modification is typically applied by directing attention away from negative stimuli and toward neutral or positive stimuli, whereas interpretation modification involves encouraging the completion of ambiguous verbal stimuli with positive content^29^.

In light of this information, it can be observed that cognitive bias modification, by facilitating changes in different stages of information processing, leads to an increase in cognitive control and flexibility, thereby serving as a protective factor against the development of depression.

### Depression and attention processes

The process of attention encompasses selecting a stimulus, focusing on it while filtering out distractions, maintaining that focus over time, and shifting focus when transitioning between tasks or stimuli. This multifaceted process forms the foundation of information processing. Attention is directed toward the goal-relevant stimulus while interacting with other cognitive functions, and it has a strong relationship with working memory^30^. Internal attention is also connected to working memory and rumination processes^31^.

The information processing process is divided into “Bottom-Up” and “Top-Down” processing^32^. Bottom-up processing occurs involuntarily based on the properties of the stimulus, while top-down processing is activated in a goal-directed manner and by motivation. Both processes are related to different brain regions; bottom-up processing is associated with the temporoparietal and inferior frontal cortices, while top-down processing is associated with the dorsolateral prefrontal cortex and anterior cingulate cortex^33,34^. Depression has been associated with an increased tendency to focus on negative information during top-down processing and with difficulties in shifting attention away from such stimuli. Sensitivity to negative stimuli is associated with impairments in prefrontal functions and reduced activity in the anterior cingulate cortex, as well as biological factors^4,35^. Research has shown that depressed individuals experience impairments in attention processes. For example, individuals with depression tend to focus more on sad facial expressions and show difficulty shifting attention toward neutral or positive ones^36^. The level of rumination plays an important mediating role between depression and attention processes^37^. Statistically significant impairments in attention have been observed in untreated depressed individuals^38^.

Research-supported impairments in attention related to depression are defined as “Attentional Bias.” Attentional bias, known as the tendency to direct attention more towards negative stimuli, has various theoretical explanations and has been the subject of numerous hypotheses and research questions^25^.

### Attentional bias and its scientific foundations

Attentional bias is defined as the tendency to focus more quickly and to a greater extent on threat-related stimuli compared to neutral or positive stimuli, and it is associated with preparedness for danger^39^. In a study conducted by Öhman et al.^39^, response times to dangerous and non-threatening stimuli were faster for dangerous stimuli, independent of other stimuli. A study by^40^ found that threat-related stimuli were effective in capturing attention, and this process occurred with automatic and parallel processing. This process was particularly evident in individuals afraid of snakes^41^. Similar findings have been supported by studies showing quick responses to dangerous stimuli in both humans and animals^42^.

The concept described by Colin^43^ as the “Cocktail Party Phenomenon” suggests that our cognitive system prioritizes stimuli related to the self. According to this phenomenon, for example, in a crowded party with many stimuli, when we hear our own name, our attention is directed to this stimulus and processed while other stimuli are suppressed. Röer and Cowan^44^ found that participants paid more attention to stimuli when they heard their own names. Similar findings in animal psychology have been supported by studies such as penguins’ ability to detect their parents’ calls and search-and-rescue dogs distinguishing their names at low decibel levels^45,46^. Actually, the response speed to self-related stimuli showed that participants responded faster to their own faces. Similarly, Orellana-Corrales et al. found that stimuli associated with the term “I” created attentional bias.

The most commonly used paradigm for assessing attentional bias is the “Dot Probe Task”. During this task, participants are shown two simultaneous stimuli, one negative and the other neutral or positive. Immediately after the presentation, both stimuli disappear, and a dot appears in the location of one of the stimuli. The participant is instructed to press the corresponding keyboard key as soon as they see the dot in that location^47^. In DPT, when assessing attentional bias, the region where the target stimulus appears is equally distributed in both directions. Additionally, the target stimulus is distributed equally among the negative and positive stimuli^48^.

There are limited studies on the neural connections involved in the DPT. During the application of DPT, the response to the target stimulus, which was paired with either a negative or neutral stimulus, was analyzed using electroencephalography measurements in two non-clinical groups with attention-avoidance and attention-vigilance tendencies. The results showed that the event-related potentials, particularly P3b, differed between the two groups for both neutral and negative stimuli. Significant differences were found between the two groups when comparing deviations and response evaluation processes related to early and late attention processes. This finding suggests that attentional bias occurs in two different forms: attention-vigilance to both negative and neutral stimuli or attention-avoidance, and brain waves vary accordingly^49^.

### Attentional bias, mood, and psychopathology

The relationship between attentional bias and mood has been examined in various samples. In a study conducted with 30 participants with anxious traits, no significant difference was found in attentional bias performance after exercise, but changes in mood and response times were observed^50^. Beck and Clark’s^51^ model suggest that anxiety is triggered by threatening stimuli, leading to attentional bias and negatively affecting an individual’s coping capacity. Hansen and Hansen^52^, using the “face in the crowd” paradigm, found that angry facial expressions were detected faster than happy ones. Similarly, Putman et al. and Maratos et al. showed that angry facial expressions create attentional bias. Lee et al. (2009) found that in a group conditioned for anxiety, the response time to conditioned facial expressions increased significantly in an emotional Stroop task.

The dynamics of attentional bias in depression are complex, yet findings consistently show a tendency to orient toward negative stimuli and away from positive stimuli^34^. Depression’s attentional bias has been explained by factors such as perception, response selection, and control of distracting stimuli^53^. Factors like the duration, severity, and rumination level of depression affect attentional bias, and it is known that there is a positive relationship between attentional bias and rumination^54–56^. The attentional bias of women with and without a history of depression. No attentional bias was observed under neutral conditions, but participants without a history of depression showed “protective bias” toward negative stimuli. Similarly, individuals diagnosed with depression exhibited more attentional bias toward sad facial expressions^23^. Depressed individuals had more difficulty diverting their attention from sad stimuli, while non-depressed individuals spent more time focusing on happy stimuli. These findings support the relationship between attentional bias in depression, sensitivity to negative stimuli, and the tendency to withdraw from positive stimuli.

### Attentional bias modification

Changing attentional bias may be effective in preventing depressive mood, ruminative thought patterns, and memory biases^30^. However, due to variables such as the duration, severity of depression, and individual cognitive structure, research findings can be contradictory^57^. This suggests the need for further research on attentional bias modification through personalized programs^49^. “Cognitive Enhancement” interventions have been found to have positive effects on verbal memory, attention processes, and working memory^11^. While attentional bias and interpretive bias modifications have been studied separately, there are also studies where these two protocols are combined. For example, a combined protocol was found to be significantly effective in obsessive–compulsive disorder^58^. However, there are also findings where no significant effects were observed in single-session applications^59^.

*Attentional bias modification* aims to redirect attention from negative stimuli to positive or neutral stimuli^60^. In attentional bias modification, paradigms such as the Dot Probe Task, which is also used in measurement, are commonly applied. In the modification process, the target stimulus predominantly appears after a positive stimulus. The presentation rate of the positive stimulus varies in research, but it generally falls between 80 and 90%. The effect of DPT has been studied in various groups and with different protocols. For example, the use of DPT in multiple sclerosis patients has been found to be effective in stress management^61^. A meta-analysis conducted with adolescents showed that cognitive bias interventions had positive effects on attentional and interpretive biases, but the effects on clinical symptoms were complex^62^. In depression treatment, the effectiveness of DPT depends on the severity of depressive symptoms. DPT is effective in mild to moderate depression, while its effectiveness decreases in severe depression^58^. Additionally, it has been found that attentional bias training is associated with rumination as a mediating variable in depressive symptoms^63^. On the other hand, there are findings indicating that DPT produces results close to placebo effects in low symptom levels^64^. The effect of stimulus type in attentional bias modification has also been investigated. Visual stimuli have been found to be more effective in social cognition, while the effect of semantic stimuli varies depending on the individual’s characteristics^60^. It is suggested that attentional bias modifications applied during the remission phase after depression treatment may be effective in reducing the risk of depression relapse^65^.

The aim of this study is to contribute to the literature by investigating whether depressive mood decreases by reducing attentional bias levels through a customized semantic stimulus set based on individuals’ cognitive biases. The study also examines the level of attentional bias as a mediating variable.

### Language and emotion

Language plays a fundamental role in conceptualizing the mental and bodily components of emotions and expressing them to the external world. The bodily sensations of emotions are categorized into words, transforming internal experiences into information that represents them^66^. Initially, emotion theorists attempted to explain the universal characteristics of emotions through categorical models^67^. However, theories such as Chomsky’s psycholinguistic approach remain limited by neuroscientific findings. Recent research shows that language is an independent system that operates in interaction with cognitive and emotional processes^68^.

Paul Ekman argued that seven basic emotions are universal, and these emotions’ facial expressions, bodily responses, and vocal forms are innate^69^. However, cross-cultural studies have shown that the conceptualization of emotions may vary across cultures. For example, the Himba ethnic group has described a smiling facial expression as “Terrifying” instead of “Happy”^70^. Lazarus’ “Cognitive Appraisal Theory” suggests that emotions are triggered by external stimuli, and these stimuli are appraised at four levels (neural, schematic, associative, conceptual)^71^. The “Constructed Action Theory” posits that emotions are concepts formed through learning, rather than being innate structures^72^. This theory suggests that the bodily components of emotions result from the combination of external sensations and conceptual knowledge^73^.

The effect of learning on emotion expression has also been explored in research. Older individuals have been found to be more successful than younger individuals in interpreting more complex facial expressions^74^. Additionally, it has been shown that language plays a crucial role in visual processing, and auditory cues increase object detection rates^75^. The relationship between language and emotions has also been studied in the context of mood disorders. For example, individuals diagnosed with depression have been shown to have difficulty distinguishing and suppressing negative emotions^76^. Studies conducted on individuals diagnosed with major depression have demonstrated that negative words remain in memory for a longer duration, and this prolongation negatively affects emotion regulation processes^77^.

It is believed that modifications through language may have an impact on mood. While the strong relationship between depression and attentional bias has been scientifically established, the findings on attentional bias modification vary depending on sample characteristics and the method of application. Attentional bias modification interventions conducted with individuals exhibiting mild to moderate depressive symptoms show promising results. Therefore, the primary aim of this study is to experimentally assess the effects of attentional bias modification on depressive mood, attentional bias, and response times to positive stimuli.

To this end, the study employed a randomized controlled design involving three distinct groups: The experimental group received attentional bias modification training, where the target stimulus was paired predominantly with positive stimuli. The placebo group followed a similar protocol, but the dot appeared equally after both positive and negative stimuli, providing no directional bias. The non-intervention group received no attentional bias training, serving as a baseline comparison.

These groups were compared to assess the differential effects of attentional bias modification on depressive affect, attentional bias levels, and reaction times. Accordingly, the following hypotheses were tested:

#### H1

*The experimental group will show a significant decrease in depressive affect levels compared to the placebo and non-intervention groups*.

#### H2

*The experimental group will show a greater reduction in reaction times to positive stimuli, indicating a greater shift in attentional bias, compared to both control groups*.

#### H3

*The Mood-State Adjective Pairs scores will significantly decrease in the experimental group, while scores in the placebo and non-intervention groups will either increase or remain unchanged*.

#### H4

*There will be no significant differences in depressive affect levels and attentional bias between the placebo and non-intervention control groups*.

## Method

### Sample

The final sample consisted of 45 participants aged between 18 and 40 years (M = 26.13, SD = 4.87). Of these, 32 participants identified as female (71.1%) and 13 as male (28.9%). Participants in the attentional-bias–modification (ABM) group had a mean age of 26.4 years (SD = 4.9; range = 19–38), those in the placebo control group averaged 25.9 years (SD = 4.8; range = 18–37), and the no-intervention control group averaged 26.1 years (SD = 5.0; range = 20–40). A one-way ANOVA confirmed that these differences were not statistically significant, *F*(2, 42) = 0.12, *p* = 0.89, indicating that age was well balanced across the three groups. Participants were recruited through online advertisements and university mailing lists. Educational backgrounds ranged from undergraduate to doctoral levels. This specific age range was chosen to avoid age-related differences in cognitive performance^78^. An initial pool of 245 volunteers (205 female, 40 male) was screened. Following clinical interviews and inclusion/exclusion criteria, only individuals with mild depressive symptoms were selected, based on Hamilton Depression Rating Scale (HDRS) scores between 8 and 16. Although the sample size was modest, the use of strict inclusion criteria and random group assignment enhances the internal validity and interpretability of findings in the context of mild depressive symptoms. Participants were excluded if they: (1) Were currently using psychiatric medications, (2) Were undergoing psychotherapy, (3) Had a history of psychiatric diagnosis or suicide attempts, (4) Had comorbid psychopathology, (5) Were experiencing grief or bereavement, (6) Had visual impairments or lacked basic computer literacy. Additionally, participants whose reaction time to negative stimuli in the Dot Probe Task exceeded that for positive stimuli (RT-negative/RT-positive > 1.00) were excluded to ensure initial presence of attentional bias.

Qualified participants were then randomly assigned to one of three groups (n = 15 in each): 1. Experimental Group (ABM intervention), 2. Placebo Group (stimuli with 50/50 positive–negative ratio) and 3. Non-intervention Control Group. Although a priori power analysis was not conducted due to the exploratory nature of the study, a minimum of 15 participants per group was based on comparable cognitive bias modification studies^23,64^.

### Procedure

This study was conducted online. In the initial phase of participant selection, structured clinical interviews were conducted by trained clinical psychologists to assess participants’ current mental health status and determine their eligibility for inclusion in the study. The primary aim of these assessments was to ensure that participants did not meet criteria for comorbid psychiatric disorders, active suicidal ideation, or ongoing psychiatric treatment, in accordance with the exclusion criteria. Then, to control for potential confounding effects arising from the online application, an attentional bias measurement was administered to a separate group (N = 20) distinct from the study participants. The group was split into two, with half completing the task online and the other half completing it face-to-face. The application was conducted consecutively but on different computers and with different internet providers. Participants’ reaction times were compared.

Participants meeting the inclusion criteria were randomly assigned to one of the three study groups: experimental, placebo, and non-intervention. (1) Experimental Group (Attentional Bias Modification—ABM): Participants completed 10 daily training sessions (each consisting of 250 trials) using a modified version of the Dot Probe Task. In this condition, the dot appeared 90% of the time behind positive words, with the aim of training attention away from negative stimuli and toward positive stimuli. Personalized positive and negative word pairs, selected by each participant, were used during the training. (2) Placebo Group (Balanced Dot Probe Task): Participants also completed 10 sessions identical in appearance and duration to the experimental group. However, in this condition, the dot appeared 50% of the time behind positive words and 50% behind negative words, thus providing no attentional training effect. This condition controlled for the mere exposure to stimuli and task repetition. (3) Non-Intervention Control Group: Participants in this group did not receive any form of Dot Probe training between the pretest and posttest. They only completed the assessments at the beginning and end of the study. All participants were blind to their group assignment, and the term “training” was used generically across groups to avoid expectation effects. The placebo and experimental tasks were identical in interface and timing to maintain consistency across conditions.

All sessions and assessments were conducted online, with additional technical and environmental controls (e.g., screen distance, posture) maintained throughout the application via TeamViewer. Clinical interviews were then conducted with eligible individuals, and the Hamilton Depression Rating Scale (HDRS) was administered (N = 145). Following the HDRS and clinical interviews, only participants with mild depressive symptoms were administered the Brief Symptom Inventory (BSI) (N = 94). Subsequently, under the supervision of the experimenter, the Mood-State Adjective Pairs List (MSAPL) and the Dot Probe Task (DPT) were administered online. Participants who did not meet the inclusion criteria during this assessment process were excluded from the study. As a result, a total of 45 participants were included in the experiment.

As the study was a single-blind experiment, participants were unaware of the group to which they were assigned. The experiment consisted of three groups: two control groups (non-intervention and placebo application) and one experimental group. Participants in the experimental and placebo groups were provided with a word list containing emotion-related words to personalize the application according to their cognitive characteristics. At this stage and during the Dot Probe Task (DPT), the Extended Turkish Affective List (GTAL), adapted from the Affective Norms for English Words (ANEW), was used^79^. Participants were asked to select words with personal significance from the GTAL that were low in valence and high in arousal. For the experimental and placebo groups, a personalized word set was created for each participant.

In the experimental condition, a total of 10 sessions were planned, each consisting of 250 stimulus sets. In these sessions, the experimental group was exposed to 90% positive and 10% negative target words. For the placebo group, 10 sessions were conducted with an equal distribution of 50% positive and 50% negative words. The second control group did not undergo any intervention between the pretest and posttest measurements.

After completing the sessions, posttest measurements were collected from all three groups using the Hamilton Depression Rating Scale (HDRS), the Mood-State Adjective Pairs List (MSAPL), and the Dot Probe Task (DPT). Pretest and posttest measurements compared participants’ depression levels, MSAPL scores, and their reaction time ratios to positive and negative stimuli in the DPT (Negative Reaction Time/Positive Reaction Time), and the results were statistically analyzed.

**Fig. 1:**
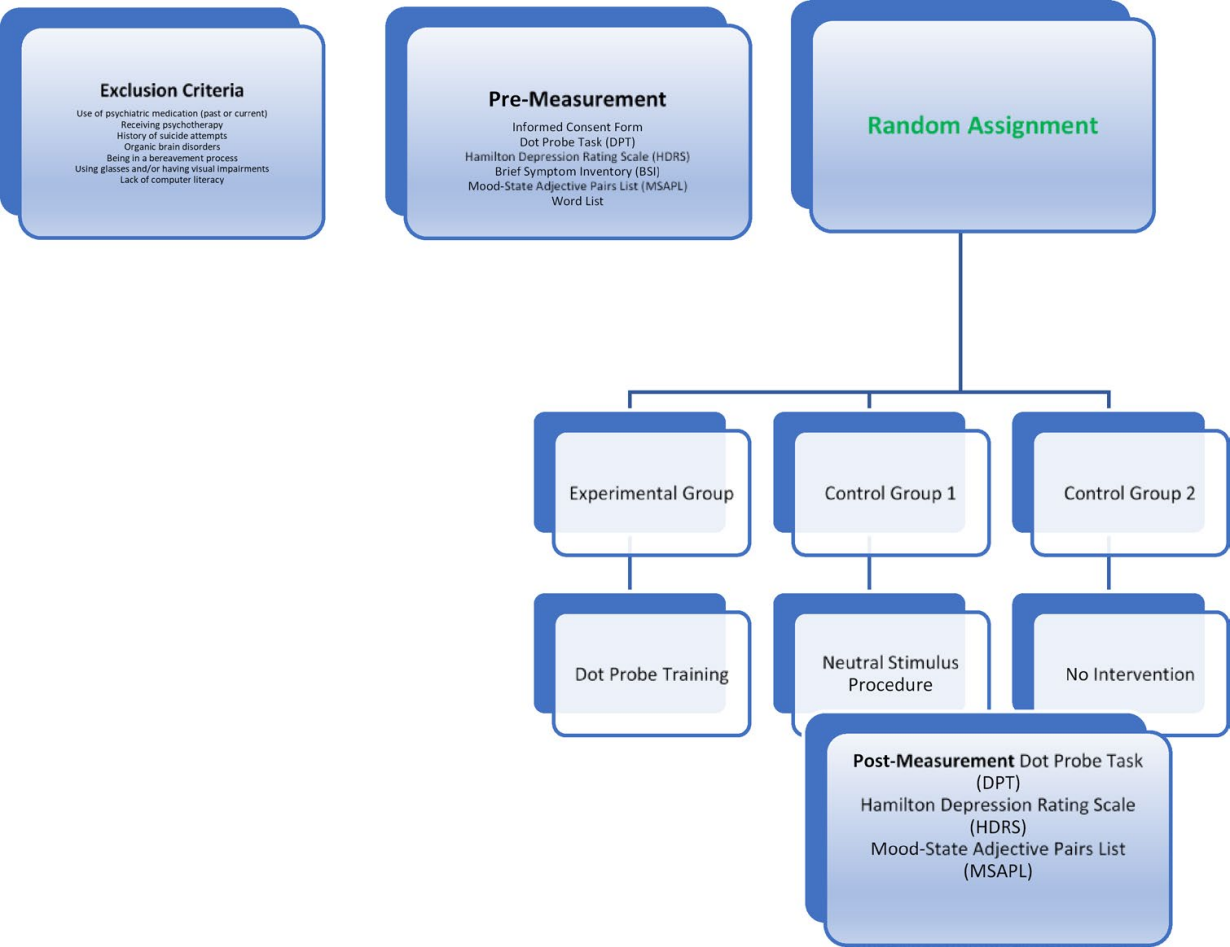
Process flowchart for the stages of the study.

During the Dot Probe Task and Dot Probe Training, the experimenter shared the computer screen via the TeamViewer program to monitor the application from start to finish. Before starting the application, a 60 cm distance between the screen and the participant was ensured, and participants were instructed to remain in a fixed position throughout the session. While the instructions were displayed on the screen in written form, the experimenter was available online to answer any questions from participants about the instructions. The process flow is illustrated in Fig. 1.

This study was approved by the Doğuş University Ethical Committee (Approval Number: E-42435178-050.06.04-36367). All methods were carried out in accordance with the relevant guidelines and regulations, including the Declaration of Helsinki for research involving human participants. Informed consent was obtained from all participants and/or their legal guardians prior to participation in the study. Identifying information has been removed to ensure participant anonymity, and no identifiable images or data have been included without explicit written consent. The data supporting the findings of this study are available upon reasonable request from Nazende Öksüz Özdemir.

### Measurement and materials

#### Demographic information form (DIF)

The Demographic Information Form includes information on participant characteristics such as participant number, the date the form was completed, age, occupation, marital status, gender, education level, eye health, and general health status.

#### Brief symptom inventory (BSI)

The Brief Symptom Inventory is a Likert-type scale consisting of 53 items. It evaluates the severity of psychological symptoms across 10 symptom groups, including 9 subscales and 1 additional scale. The original inventory was developed by Derogatis in 1992, and its Turkish adaptation was conducted by Hisli Şahin and Durak in 1994. To determine reliability, a Cronbach’s alpha analysis revealed item-total correlation coefficients ranging from 0.21 to 0.78, with an overall reliability coefficient of 0.97. The BSI comprises nine primary symptom dimensions and one global index. These subscales are: (1) Somatization—measures distress arising from perceived bodily dysfunction, (2) Obsessive–Compulsive—assesses intrusive thoughts and compulsive behaviors, (3) Interpersonal Sensitivity—reflects feelings of personal inadequacy and inferiority, (4) Depression—captures symptoms such as dysphoric mood, loss of interest, and hopelessness, (5) Anxiety—measures nervousness, tension, and panic symptoms, (6) Hostility—indicates anger, irritability, and aggression, (7) Phobic Anxiety—captures persistent fears related to specific situations or objects, (8) Paranoid Ideation—assesses suspiciousness and paranoid thinking, (9) Psychoticism—reflects symptoms ranging from social alienation to psychotic-like experiences. The additional scale, or Global Severity Index (GSI), serves as an overall measure of psychological distress and symptom intensity.

#### Hamilton depression rating scale (HDRS)

The Hamilton Depression Rating Scale, developed by Hamilton in 1960, underwent a validity and reliability study for Türkiye by Akdemir and colleagues in 1996. The scale aims to determine the severity and symptom patterns of depression. In the structured form of the HDRS, the questions to be delivered to participants are predetermined (Akdemir, Örsel, Dağ, Türkçapar, İşçan, & Özbay, 1996), eliminating any potential bias caused by how the questions are conveyed. In this study, the internal consistency coefficient (Cronbach’s alpha) of the scale was found to be 0.75^80^.

#### Mood-state adjective pairs list (MSAPL)

The Mood-State Adjective Pairs List was used in this study as a self-report scale to assess depressive mood. The reliability study of the scale utilized the Beck Depression Inventory^81^. The MSAPL aims to evaluate the interaction of emotions and cognition, primarily in cognitive psychology and other related fields. The MSAPL is composed of three subscales, each designed to assess a specific dimension of affective and cognitive processing: Cognitive Subscale (21 items): Measures thought-related aspects of mood, such as *focused–distracted*, *clear–confused*, and *confident–doubtful*. Emotional Subscale (19 items): Assesses emotional states such as *happy–sad*, *relaxed–tense*, and *content–irritated*. Cognitive-Emotional Mixed Subscale (32 items): Combines both emotional and cognitive dimensions, including pairs like *motivated–unmotivated*, *interested–indifferent*, and *hopeful–hopeless*. This Likert-type scale consists of 72 adjective pairs rated on a scale of 1 to 7. Participants were asked to rate each adjective pair according to how well it matched their current mood, with 1 indicating the most positive and 7 indicating the most negative. Lower scores indicate a reduction in negative mood^82^. The reliability study determined the internal consistency coefficient (Cronbach’s alpha) of the scale to be 0.94.

#### Word lists used in the experimental and placebo groups

The database created through affective word rating, known as the Affective Norms for English Words (ANEW), was expanded and adapted to Turkish in 2019 as the Extended Turkish Affective List (GTAL). In the adaptation study, words were rated on arousal and valence on a scale from 0 to 9^79^. In this study, participants in the experimental and placebo groups were given a list composed of words rated as low in valence (0–3), meaning they evoked negative feelings, and high in arousal (6–9), meaning they carried strong emotional content, based on the GTAL. This list identified words with negative content relevant to the participants. Additionally, words rated high in both valence (6–9) and arousal (6–9) were provided to identify positive content. Participants were asked to mark the words on the list that resonated with them.

#### Dot probe task (DPT)

The Dot Probe Task, developed by MacLeod, Mathews, and Tata in 1986, is a paradigm used to measure and modify attention processes and attentional bias^83^. In this study, the “OpenSesame 3.3.9” software, commonly used in psychology, neuroscience, and experimental economics research, was employed. Three different interface workflows were prepared in OpenSesame: a test phase (Dot Probe Task-DPT), an experimental phase (Dot Probe Training-DPTT), and a control application (Dot Probe Task-Placebo). Word pairings were uploaded into this interface, and dot directions were determined based on appropriate percentages.

During interventions with participants in the experimental and control groups, words selected by each participant from the provided word list were used.

The first phase of the application displayed an “X” symbol in the center of the screen, which remained visible for 1000 ms before disappearing. Participants focused on this symbol, and in the second phase, a pair of words was displayed 13 cm to the left and right of the “X” symbol. One stimulus in the pair consisted of negative content, while the other contained positive content. The stimuli were displayed for 500 ms and then disappeared, followed by the appearance of a dot on one side (left or right). Participants were instructed to press the corresponding left or right arrow key on the keyboard as soon as they saw the dot. Reaction times were recorded automatically throughout the test and intervention. If a response was made in the wrong direction, the screen remained unchanged, and the experiment did not progress until the correct response was provided. Meanwhile, the reaction time recording continued.

**Fig. 2:**
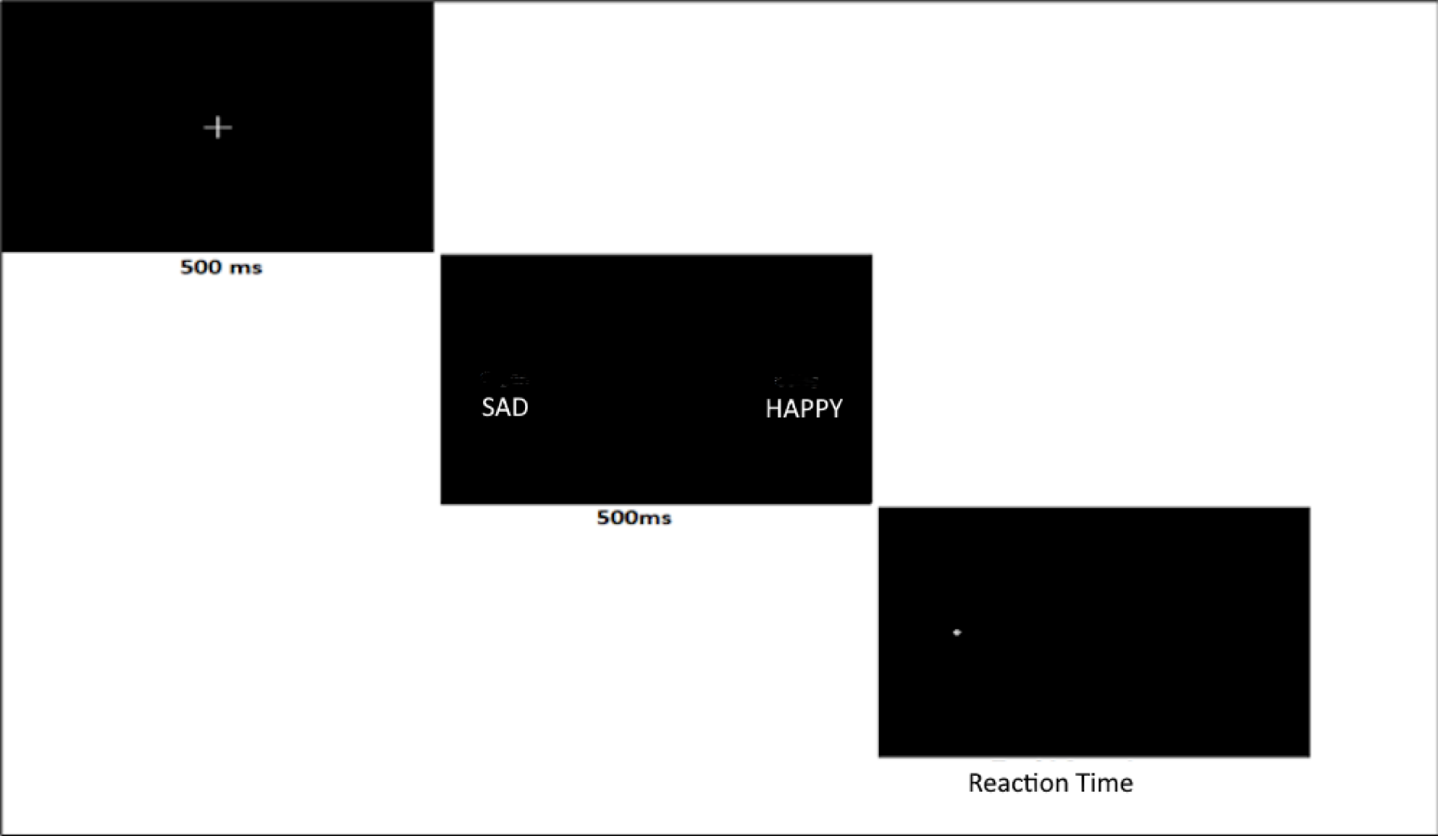
Dot probe task.

The distribution of positive and negative words and the location of the dot in the DPT were balanced equally between the left and right sides. In other words, the ratio of dots appearing after negative words and positive words was set at 50%. Additionally, the dot was shown equally across the two locations (Fig. 2).

#### Dot probe training (DPTT)

This training, designed to redirect attention, consisted of 10 sessions, each involving 250 repetitions, conducted over 10 days. While the planned number of sessions varies across studies, a review of the literature determined the average session count to be approximately 10.9^23^. The procedure was identical to the Dot Probe Task (DPT); however, in this case, the dot was paired with positive words 90% of the time. This aimed to redirect attention away from negative stimuli and toward positive stimuli.

In one of the control group procedures, participants underwent a similar protocol of 10 sessions with 250 repetitions each. However, the primary difference in this procedure was that attention was not directed toward any target stimulus. The ratio of dots paired with positive and negative words was equally distributed at 50%.

## Results

### Data analysis

A general assumption for normal distribution is having a sample size of at least 30 (n ≥ 30). The fundamental premise of this theorem is that a sample size of n ≥ 30, selected randomly from the population, ensures a normal distribution^84^. It also implies that the assumption of normal distribution may not be met when the sample size is smaller than 30 (n < 30). The assumption of normal distribution was evaluated based on skewness and kurtosis values. A range of − 2 to + 2 for skewness and kurtosis indicates normal distribution^85^. Therefore, parametric analyses were chosen for this study.

One-way ANOVA was performed to analyze the differences between groups for the Hamilton Depression Rating Scale, Mood-State Adjective Pairs List, Dot Probe Task, and Brief Symptom Inventory. To examine the combined effect of groups with pretest and posttest results of the Hamilton Depression Rating Scale, Mood-State Adjective Pairs List, and Dot Probe Task, Two-Way Mixed ANOVA was used. All analyses in this study were conducted with a 95% confidence interval, and a *p*-value of 0.05 was set as the reference threshold. The distribution of demographic variables is provided in Table 1.

**Table 1:** Distribution by demographic variables.

	Group					
	Experimental group		Control group 1 (Placebo)		Control group 2 (No intervention)	
	n	%	n	%	n	%
Gender						
Female	15	93.8	13	92.9	14	93.3
Male	1	6.3	1	7.1	1	6.7
Total	16	100.0	14	100.0	15	100.0
Education						
Undergraduate	12	75.0	10	71.4	8	53.3
Master’s Degree	3	18.8	4	28.6	7	46.7
Doctorate	1	6.3	0	0.0	0	0.0
Total	16	100.0	14	100.0	15	100.0
Marital status						
Married	9	56.3	6	42.9	6	40.0
Single	7	43.8	8	57.1	9	60.0
Total	16	100.0	14	100.0	15	100.0

When comparing the group variable across Anxiety, Depression, Hostility, Somatization, Negative Self, Global Severity Index, Total Symptom Index, and Symptom Distress Index, no significant differences were found in the mean scores obtained from the scales (*p* > 0.05; Table 2).

**Table 2:** Comparison of scores obtained from the brief symptom inventory by group variable.

	n	${\overline{\text{X}}}$	Ss	Var. K	K.T	Sd	K.O	F	*p*
Anxiety									
Experimental group	16	1.17	0.70	Between groups	0.32	2	0.16	0.34	0.713
Control group 1 (Placebo)	14	0.97	0.74	Within groups	19.68	42	0.47		
Control group 2 (No intervention)	15	1.05	0.62	Total	20.00	44			
Total	45	1.07	0.67						
Depression									
Experimental group	16	2.06	0.85	Between groups	0.02	2	0.01	0.02	0.984
Control group 1 (Placebo)	14	2.01	0.87	Within groups	30.07	42	0.72		
Control group 2 (No intervention)	15	2.03	0.82	Total	30.09	44			
Total	45	2.03	0.83						
Hostility									
Experimental group	16	1.44	0.86	Between groups	1.30	2	0.65	0.92	0.406
Control group 1 (Placebo)	14	1.03	0.81	Within groups	29.65	42	0.71		
Control group 2 (No Intervention)	15	1.30	0.85	Total	30.95	44			
Total	45	1.26	0.84						
Somatization									
Experimental group	16	0.90	0.63	Between groups	0.28	2	0.14	0.32	0.729
Control group 1 (Placebo)	14	0.83	0.80	Within groups	18.35	42	0.44		
Control Group 2 (No Intervention)	15	0.71	0.55	Total	18.62	44			
Total	45	0.82	0.65						
Negative self									
Experimental group	16	1.43	0.95	Between groups	0.86	2	0.43	0.56	0.576
Control group 1 (Placebo)	14	1.25	0.74	Within groups	32.22	42	0.77		
Control group 2 (No Intervention)	15	1.59	0.90	Total	33.08	44			
Total	45	1.42	0.87						
Global severity index									
Experimental group	16	1.49	0.83	Between groups	0.49	2	0.25	0.43	0.651
Control GROUP 1 (Placebo)	14	1.24	0.73	Within groups	23.80	42	0.57		
Control group 2 (No intervention)	15	1.39	0.69	Total	24.30	44			
Total	45	1.38	0.74						
Total symptom index									
Experimental group	16	34.00	12.41	Between groups	109.64	2	54.82	0.39	0.680
Control group 1 (Placebo)	14	30.43	12.35	Within groups	5905.16	42	140.60		
Control group 2 (No intervention)	15	31.13	10.73	Total	6014.80	44			
Total	45	31.93	11.69						
Symptom distress index									
Experimental group	16	2.18	0.68	Between groups	0.56	2	0.28	0.79	0.462
Control group 1 (Placebo)	14	1.99	0.61	Within groups	14.92	42	0.36		
Control group 2 (No intervention)	15	2.26	0.48	Total	15.47	44			
Total	45	2.15	0.59						

*p < 0.05 one-way analysis of variance (ANOVA).

The interaction between participants’ Hamilton Depression Rating Scale pretest and posttest scores and the group variable was examined. To evaluate this interaction, a Two-Way Mixed Analysis of Variance (ANOVA) was conducted. In the initial stage of the analysis, the equality of covariance matrices (Box’s M test) and the homogeneity of group variances (Levene’s test) were assessed. Both assumptions were found to be satisfied (*p* > 0.05). It was determined that the effects of the measurement time points and group membership on Hamilton Depression Rating Scale scores were statistically significant (F(2, 42) = 6.73, *p* < 0.05), as shown in Table 3. Post hoc Tukey test indicated that the experimental group’s posttest scores on the Hamilton Depression Rating Scale were significantly lower than those of the no-intervention control group (*p* < 0.05).

**Table 3:** Comparison of group variables and hamilton depression rating scale pretest and posttest scores.

	S.S	df	M.S	F	p	η^2^
Ham	171.47	1.00	171.47	29.62	< 0.001*	0.41
Ham * Group	77.99	2.00	38.99	6.73	0.003*	0.24
Error	243.17	42.00	5.79			

*p < 0.05 two-way mixed analysis of variance.

The interaction between participants’ Mood-State Adjective Pairs List pretest and posttest scores and the group variable was examined. To evaluate this interaction, a Two-Way Mixed Analysis of Variance (ANOVA) was conducted. In the initial stage of the analysis, the equality of covariance matrices (Box’s M test) and the homogeneity of group variances (Levene’s test) were assessed, and both assumptions were found to be satisfied (p > 0.05). Post hoc Tukey analysis revealed that the experimental group showed significantly lower posttest scores on the Mood-State Adjective Pairs List compared to the no-intervention control group (p < 0.05). It was determined that the effects of measurement time points and group membership on Mood-State Adjective Pairs List scores were statistically significant (F(2, 42) = 6.12, *p* < 0.05), as shown in Table 4.

**Table 4:** Comparison of group variables and mood-state adjective pairs list pretest and posttest scores.

	S.S	df	M.S	F	p	η^2^
DDSÇ	19,826.75	1.00	19,826.75	11.97	0.001*	0.22
DDSÇ * Gorup	20,274.95	2.00	10,137.47	6.12	0.005*	0.23
Error	69,573.21	42.00	1656.50			

*p < 0.05 two-way mixed analysis of variance.

The results of the Two-Way Mixed Analysis of Variance (ANOVA) conducted to determine the combined effect of the group variable and the Dot Probe Task pretest and posttest scores are presented in Table 5. The interaction between participants’ Dot Probe Task pretest and posttest scores and the group variable was examined. To evaluate this interaction, a Two-Way Mixed Analysis of Variance (ANOVA) was performed. In the initial stage of the analysis, the equality of covariance matrices (Box’s M test) and the homogeneity of group variances (Levene’s test) were assessed, and both assumptions were found to be satisfied (*p* > 0.05). Post hoc Tukey analysis indicated that the experimental group showed a significantly greater improvement in attentional bias scores (measured via Dot Probe Task) compared to the no-intervention control group (*p* < 0.05).

**Table 5:** Comparison of group variables and dot probe task pretest and posttest scores.

	S.S	df	M.S	F	p	η2
NYBG	0.00	1	0.00	0.91	0.344	0.02
NYBG * Group	0.03	2	0.01	5.18	0.010*	0.20
Error	0.10	42	0.00			

*p < 0.05 two-way mixed analysis of variance.

**Fig. 3:**
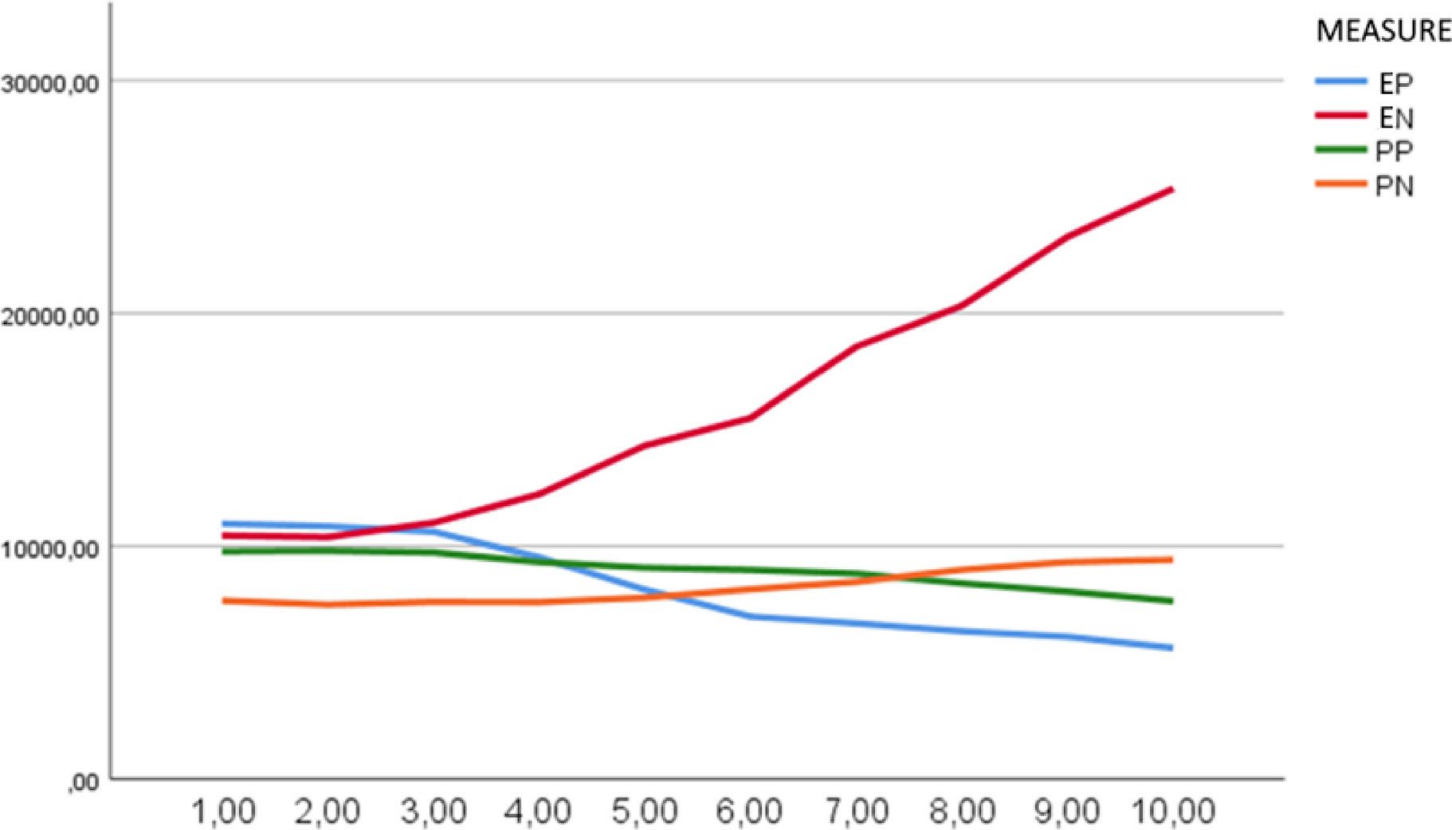
Comparison of reaction times in dot probe training between experimental and placebo groups. The X-axis represents the number of sessions and the Y-axis represents reaction time in milliseconds. EP = Experimental Group – Positive Stimuli, EN = Experimental Group – Negative Stimuli, PP = Placebo Group – Positive Stimuli, PN = Placebo Group – Negative Stimuli.

The reaction time ratios measured during the sessions of the Dot Probe Training applied to the experimental and placebo groups are illustrated in Fig. 3. According to this figure, the reaction times to positive stimuli decrease proportionally over time in both groups, while reaction times to negative stimuli increase. However, it was observed that the change in reaction times occurred earlier in the experimental group compared to the placebo group.

## Discussion

In terms of attentional bias, the experimental group showed a reduction in attentional bias levels, while notable changes were also observed in the placebo group. This finding indicates that attentional bias modification was effective in this study. While the primary objective of the research was to examine whether attentional bias modification could reduce depressive affect, the findings showed that the most consistent and significant change occurred in attentional bias itself.

Hypothesis 1 was supported by the findings, demonstrating that the attentional bias levels of the experimental group decreased, while notable changes were also observed in the placebo group, the magnitude and timing of these changes were less pronounced compared to the experimental group, and no significant changes were observed in the no-intervention group. This result confirms the effectiveness of attentional bias modification.

Hypothesis 2 was also supported by the findings. The reduction in attentional bias within the experimental group aligns closely with the findings in the existing literature^63^. However, differences observed in the placebo group are addressed in the discussion section of the study.

Hypothesis 3 was partially supported by the findings. While a decrease in total scores on the Mood-State Adjective Pairs List (MSAPL) was observed in the experimental group, a significant decrease was also identified in the placebo group. However, no significant changes were found in the no-intervention group. According to the subscale analysis of MSAPL, which measures depressive affect, the cognitive subscale scores in the experimental group were significantly different compared to the other groups. This difference was consistent with the observed reduction in attentional bias. However, no significant differences were found among the other subscales.

Hypothesis 4 was supported with respect to attentional bias but not depressive affect levels. Significant differences were found among the control groups concerning depressive affect levels. This indicates that Hypothesis 4 was supported only in terms of attentional bias, but not for depressive affect.

### Discussion of pretest–posttest findings regarding depressive affect symptom levels

Significant changes in depressive affect were observed in both the experimental and placebo groups, but not in the no-intervention group. While the changes in the experimental group were consistent with the hypothesis, the improvements in the placebo group were not anticipated.

Previous findings suggest that attentional bias modification (ABM) is more effective at higher symptom levels^48^, though contradictory results also exist^57,63^. Given that participants in the present study had mild symptoms, these improvements may have been influenced by external factors or non-specific therapeutic effects.

The nature of placebo conditions in Dot Probe Task (DPT) studies varies, with some using neutral pairs and others using emotionally valenced pairs^86,87^. In our study, using positive–negative stimuli in the placebo group may have inadvertently created an active condition, possibly introducing a learning effect^88^. This could partially explain the unexpected change observed in this group.

Studies using neutral stimuli in the placebo condition tend to show clearer differences between experimental and control groups^89^, while emotionally neutral placebo conditions have occasionally yielded improvements^90^. These inconsistencies highlight the importance of better defining and standardizing control conditions in ABM research^91^.

Analysis of the Mood-State Adjective Pairs List (MSAPL) revealed that only the experimental group showed significant improvement in the Cognitive Subscale, suggesting cognitive changes that align with attentional bias findings. The Emotional and Cognitive-Emotional subscales, however, showed improvement in both the experimental and placebo groups, while no change was observed in the no-intervention group.

These results raise the possibility that cognitive improvements may precede or contribute to affective improvements. This aligns with existing theories that link attentional biases and cognitive control with emotion regulation and affective processing^8^.

Moreover, impairments in attention and memory processes are known to share neural mechanisms with mood disorders^92^, and attentional bias is linked to brain regions such as the amygdala, insula, and dorsolateral cortex. These cognitive-emotional interactions are critical for understanding the development and maintenance of depression, and targeting them may have preventive value.

This study contributes to this discussion by providing initial evidence that cognitive changes through ABM may influence affect. Future studies should investigate this relationship more closely, with greater methodological precision and longitudinal data.

### Discussion on attentional bias level variable

According to the analysis of attentional bias measurements, a decrease in attentional bias (response time to negative stimuli/response time to positive stimuli) was observed in the experimental group, whereas the placebo group showed a similar trend of change over time, though a lesser degree and with delayed onset compared to the experimental group. No significant change was observed in the no-intervention group. The practice effect was addressed by differentiating the words used in the pre- and post-test measurements from those used during training. Consequently, the observation of significant differences in pre- and post-test measurements of the Dot Probe Task (DPT) exclusively in the experimental group suggests the absence of a practice effect influencing the results.

Some studies on attentional bias modification have indicated that significant changes in attentional bias can occur even after a single session of intervention^93^. To examine the effect of attentional bias modification on depression, this study developed personalized stimulus protocols tailored to the cognitive structures of participants, consistent with the literature emphasizing the efficacy of individualized interventions^94^.

This study included an experimental group, a no-intervention control group, and a placebo group with an equal distribution of positive and negative stimuli. The significant changes observed exclusively in the Cognitive Subscale of the Mood-State Adjective Pairs List for the experimental group support the findings of cognitive changes and reductions in attentional bias.

In recent years, brain imaging techniques such as fMRI have been used to investigate the effects of attentional bias modification. A study conducted in 2020 observed decreased activity in brain regions associated with ruminative thinking in the experimental group^95^. Conversely, in a study focusing on anxiety, although no significant differences were found between the experimental and control groups following attentional bias modification, increased gray matter activity in certain brain regions was detected. This finding underscores the importance of utilizing more advanced tools for assessing attentional bias^96^.

When examining the mean reaction times of the experimental and placebo groups, both groups exhibited prolonged response times to negative stimuli and shorter response times to positive stimuli. However, this change occurred more rapidly in the experimental group, supporting the differences observed in the post-test measurements of the Dot Probe Task (DPT).

## Conclusion

### Contributions of the study

Preventive mental health interventions aiming to prevent the development of psychopathology have gained increasing importance over time. This study contributes to the development of a preventive mental health intervention program aimed at mitigating the risk of depression. Considering the heightened risk of depression during early and middle adulthood^63^, this research offers a significant contribution to the literature by focusing on the prevention of depression among participants aged 20–40.

The effects of attentional bias modification (ABM) on depressive symptom severity show variability in the literature. One contributing factor to this variability is the lack of distinction between depressive affect and accompanying anxiety symptoms, as well as the inclusion of threat-related stimuli in protocols^48^. This study controlled for such confounding variables by excluding participants with comorbid diagnoses, thereby enabling a focused investigation of depressive affect.

Attentional biases are shaped by personal experiences and align with an individual’s cognitive framework, typically involving negative or threatening stimuli^94^. In light of this, the stimuli used in the study were personalized, aiming to provide novel contributions to the scientific literature. While anxiety-based psychopathologies tend to show attentional biases towards threat-related stimuli, depressive symptoms are associated with biases towards sadness-related stimuli^97^. Consequently, the word sets used in the study were created with sadness and happiness content to align with these tendencies.

In the assessment of depression, the importance of using both clinician-administered scales and self-report measures has been emphasized in the literature^98^. To ensure a reliable evaluation, both types of scales were employed in this research.

The findings indicate that attentional bias intervention effectively reduced attentional bias levels in the experimental group. Investigating and developing such interventions for their potential to support psychotherapy processes is considered promising.

In recent years, studies have explored the feasibility of transforming attentional bias modification into mobile applications across various samples^61^. This research also aimed to develop a remote, accessible technique to facilitate depression intervention, marking it as the first known study to examine depressive affect through a remote application. In this respect, it holds the potential to contribute to scientific research and provide a foundation for future studies.

### Limitations of the study

Although the study targeted an age range considered at risk for depression, one limitation is the inability to compare findings across different age groups. Additionally, the long-term effects of attentional bias modification (ABM) were not investigated due to the potential influence of confounding variables and the dynamic nature of affect over time. As a result, the longitudinal impact of the intervention remains unexplored.

The impact of word-based ABM on depressive symptoms shows an inconsistent profile when compared to visual stimuli; while some studies report the effectiveness of word-based training^63^, others suggest greater efficacy with visual stimuli^65^. This study exclusively used a word-based stimulus set due to the absence of a standardized set incorporating visual cues associated with depressive affect. The lack of visual stimuli can therefore be considered a limitation.

Additionally, variability in findings may stem from factors such as the duration of the intervention (a meta-analysis on anxiety disorders found that extending training duration up to 50 min increased effectiveness^99^, the frequency of sessions, and the content of the words used^64^.

Recent studies have utilized advanced intervention and measurement tools, such as eye-tracking technology and virtual reality headsets^64^. However, due to the remote nature of the study conducted during the COVID-19 pandemic and financial constraints, these advanced tools were not employed. This limitation suggests that the findings may differ if such high-level measurements were used.

Furthermore, many studies involving the Dot Probe Task (DPT) face limitations regarding sample size^63^. Similarly, the limited sample size in this study constrains the generalizability of its findings.

### Recommendations for future research

Developing a mobile application capable of influencing affect and attentional bias is now feasible with advancements in technology. This study aimed to develop a supportive, preventive, cost-effective, and easily accessible intervention. The findings suggest that attentional bias can be reduced through such applications, offering promising implications. Interventions targeting attentional processes to enhance cognitive flexibility and reduce depressive symptoms could provide significant contributions.

Research on “Cognitive Remediation” interventions has shown effectiveness in conditions like depression, anxiety disorders, addiction, and even schizophrenia^58,90,100^. Future studies evaluating different intervention protocols are deemed essential. The existing literature on depressive symptoms and depression treatment is promising. Combining attentional bias modification (ABM) with interventions targeting other cognitive processes in future studies could offer further insights.

Since attentional bias in depression involves heightened sensitivity to negative stimuli and inhibition of positive stimuli^34^, this study utilized a combination of negative and positive stimuli. However, future protocols could focus on comparing different stimulus pairings, such as neutral stimuli paired with positive or negative stimuli, particularly in subclinical populations with depressive affect. Additionally, extending ABM interventions over longer periods with repeated training sessions could increase the longevity of their effects.

Research indicates that ABM may produce different outcomes depending on the severity of depressive symptoms. Comparative studies involving individuals with mild depressive symptoms and those with moderate-to-severe depression using the same stimulus sets and larger samples could contribute to the literature. Follow-up studies, as well as comparisons involving samples with depressive symptoms co-occurring with anxiety, are also essential. Examining variables known to contribute to depression, such as emotional regulation and rumination, as mediators in research protocols could provide valuable insights.

There are limited studies investigating biosignals associated with attentional bias and identifying neural connections^101^. Research utilizing fMRI to explore these connections would be highly valuable. Additionally, the development of self-report scales and clinician-administered tools to evaluate attentional bias, particularly for subclinical populations, is needed in both international and national literature^102^. Such tools could enhance the validity of findings by assessing cognitive impairments associated with depression.

This study did not include a reward-based intervention alongside attentional redirection. Research involving reinforcement schedules in similar and diverse sample groups could contribute to the literature. Considering the existence of various ABM protocols^103^, studies comparing these protocols with the Dot Probe Task would also provide valuable insights. Meta-analyses of ABM and other cognitive bias modification interventions remain limited^62^. Expanding meta-analyses with standardized protocols would enhance the reliability of findings and provide a more comprehensive understanding of these interventions.

Finally, transforming cognitive bias interventions, which are considered to play a protective role in the development and progression of depression, into accessible forms such as mobile applications could greatly benefit future research and practical applications.

### Conclusion

This study aimed to reduce attentional bias and depressive affect levels through attentional bias modification by comparing an experimental group with two control groups. In the initial stage, online and face-to-face implementations were compared, and the sample for the study was established thereafter. For pre-measurement, the Brief Symptom Inventory was applied, followed by clinical interviews. The effects of attentional bias modification were examined using the Hamilton Depression Rating Scale, the Mood-State Adjective Pairs List, and the Dot Probe Task. Parametric techniques were used to analyze the findings.

The results indicated that attentional bias levels decreased in the experimental group but remained unchanged in the two control groups. This supports the part of Hypothesis 1 related to attentional bias. Depressive affect levels decreased in both the experimental group and the placebo group but showed no change in the non-intervention control group. Since Hypothesis 1 also predicted a reduction in depressive affect specifically in the experimental group, this aspect of the hypothesis was only partially supported. . Additionally, the Mood-State Adjective Pairs List findings showed cognitive improvements exclusively in the experimental group. Both the placebo and experimental groups demonstrated increased reaction times to positive stimuli and prolonged reaction times to negative stimuli, but this change occurred more rapidly in the experimental group. In other words, while both groups benefited from the intervention, the benefits were more pronounced and occurred more quickly in the experimental group.

However, the study has certain limitations. The inclusion of positive–negative stimulus pairs in the placebo condition could be considered a limitation, and future research may benefit from using neutral stimulus sets. The inability to compare findings across different age groups and the absence of a follow-up study are additional limitations. The literature contains limited research comparing different types of stimuli, and this study did not include a comparison of visual and verbal stimuli. Utilizing advanced measurement tools such as eye-tracking techniques and fMRI in attentional bias modification studies could yield more definitive insights. Repeating the study with larger samples is also critical for broader generalization.

Developing a mobile application to effectively target attentional bias and depressive affect has become feasible with advancements in modern technology. Attentional bias modification studies suggest promising opportunities for reducing these biases through such technological tools. Future research could explore protocols integrating multiple cognitive bias modification techniques and reinforcement schedules. Studies comparing participants with varying levels of depressive affect could also enrich the existing literature.

This study aimed to create a supportive and preventive short, cost-effective, and accessible intervention for individuals aged 20–40, a demographic at high risk for depression. The single-blind design and exclusion of comorbidity ensured control over confounding variables. Participants’ lack of knowledge regarding the intervention eliminated the expectancy effect.

This study is the first known research to investigate depressive affect using remote applications in the field. As such, it holds potential to contribute significantly to the development of time-efficient and accessible techniques for addressing depression in a scalable and practical manner.

## Data Availability

The data supporting the findings of this study are available upon reasonable request from Nazende Öksüz Özdemir.
